# Two new species of *Begonia, B. moneta* and *B. peridoticola* (Begoniaceae) from Sabah, Malaysia

**DOI:** 10.1186/s40529-015-0087-5

**Published:** 2015-04-10

**Authors:** Ching-I Peng, Che-Wei Lin, Rimi Repin, Yoshiko Kono, Wai-Chao Leong, Kuo-Fang Chung

**Affiliations:** 1 Herbarium (HAST), Biodiversity Research Center, Academia Sinica, Nangang 115, Taipei, Taiwan; 2 Division of Botanical Garden, Taiwan Forestry Research Institute, No. 53, Nan-Hai Road, Taipei, 100 Taiwan; 3 Sabah Parks, Kota Kinabalu, 88806 Sabah, Malaysia; 4 School of Forestry and Resource Conservation, National Taiwan University, Taipei, 106 Taiwan

**Keywords:** Begonia moneta, Begonia peridoticola, Borneo, Chromosome number, Malaysia, New species, Sabah, Sect. Baryandra, Sect. Petermannia

## Abstract

**Background:**

Mount Kinabalu, reknowned for its high biodiversity and endemism, is a National Park in the State of Sabah on the northern end of the island of Borneo. Every year many visit the higher part of the Kinabalu National Park, while most lowland forests in the Park are under-explored. Two unknown species of *Begonia* were collected from a peridotic (ultramafic) cliff in the Kinabalu National Park at ca. 400 m elevation.

**Results:**

The two species are named *B. moneta* C.-I Peng, Rimi & C. W. Lin and *B. peridoticola* Rimi, C.-I Peng & C. W. Lin. *Begonia moneta* (sect. *Baryandra*) is similar to *B. gueritziana* Gibbs, a widespread species of the same section in Borneo, differing in the peltate (vs. basifixed) leaves and the smaller flower parts. Also, their chromosome numbers are different (*B. moneta*, 2*n* = 30; *B. gueritziana*, 2*n* = 28). The peltate and succulent foliage of *B. moneta* is also reminiscent of *B. burttii* Kiew & S. Julia and *B. payung* S. Julia & Kiew, both of sect. *Reichenheimia*, from Sarawak. *Begonia moneta* is distinct from the two species in having branched (vs. entire) placental lamellae. Additionally, *B. moneta* differs from *B. burttii* in having 4 (vs. 5) tepals in pistillate flowers and markedly unequal (vs. equal) fruit wings. *Begonia moneta* differs from *B. payung* in the smaller leaves and conspicuously winged (vs. wingless) capsules. *Begonia peridoticola* (sect. *Petermannia*) resembles *B. punchak* Kiew & S. Julia from limestone areas in Kuching Division, Sarawak, differing in the entire leaf margin (vs. distantly dentate), much larger capsular wings (8–11 mm vs. 2–3 mm wide) and yellow, spiral (vs. crimson, U-shaped) styles.

**Conclusion:**

A careful study of the herbarium materials and literature supports the recognition of the two new species. Detailed descriptions, line drawings, color plates, chromsome data, foliar SEM observations and comparisons with phenetically similar species are provided to aid in identification.

**Electronic supplementary material:**

The online version of this article (doi:10.1186/s40529-015-0087-5) contains supplementary material, which is available to authorized users.

## Background

Mount Kinabalu, reknowned for its high biodiversity and endemism, is a National Park in the State of Sabah on the northern end of the island of Borneo (Somaweer [2007]). Every year many visit the higher part of the Kinabalu National Park, while most lowland forests in the Park are under-explored. Several new species of *Begonia* were documented from Sabah in recent years (Reza & Kiew [1998]; Beaman et al. [2001]; Kiew [1998]; [2001]; Kiew & Tan [2004]). In continuation of our recent taxonomic studies on Asian *Begonia* (e.g. Chung et al. [2014]; Ding et al. [2014]; Lin & Peng [2014], Lin et al. [2014a], [b]; Lin et al. [2015]; Nakamura et al. [2013]; Peng et al. [2013], [2014a], [b], [c]; Rubite et al. [2013], [2014]), we report the discovery of two new species of *Begonia, B. moneta* and *B. peridoticola*, that co-occur on a peridotic (ultramafic) cliff in the Kinabalu National Park at ca. 400 m elevation. The two species belong to different sections, and no sign of natural hybridization was apparent locally.

## Methods

### Chromosome preparations

Somatic chromosomes of *Begonia moneta* were examined using root tips from plants of the type collection. The methods of pretreatment, fixation and staining for chromosome observations followed Peng et al. ([2014a]). Classification of the chromosome complements based on centromere position at mitotic metaphase follows Levan et al. ([1964]). Voucher specimen (*Peng 22343*) is deposited in HAST.

### Cryo scanning electron microscopy

Fresh leaves of *Begonia moneta and B. peridoticola* were dissected and attached to a stub. The samples were frozen with liquid nitrogen slush, then transferred to a sample preparation chamber at −160°C and etched for 15 min at −85°C. After etching, the temperature droped to −130°C for sample fracturing and coating. After coating, the samples were transferred to the SEM chamber and observed at −160°C with a cryo scanning electron microscope (FEI Quanta 200 SEM/Quorum Cryo System PP2000TR FEI). Voucher specimens (*Begonia moneta*: *Peng 22344*; *B. peridoticola*: *Peng 22343*) are deposited at HAST.

## Results and discussion

### Species description

**1.** *Begonia moneta* C.-I Peng, Rimi & C. W. Lin, *sp. nov.* **-TYPE:** MALAYSIA. Borneo. Sabah. Kota Marudu District: Kinabalu Park – Serinsim substation, Bat Cave Trail, elev. *ca.* 300–350 m. Collected on 13 November 2009; type specimens pressed from plants brought back from the field and cultivated in the experimental greenhouse. *Ching-I Peng 22344-A,* with *Kuo-Fang Chung, Wai-Chao Leong & Rimi Repin* (holotype: SNP; isotypes: A, E, HAST, KEP, SAN, TAIF).

銀幣秋海棠Figures 1 and 2.

**Figure 1:**
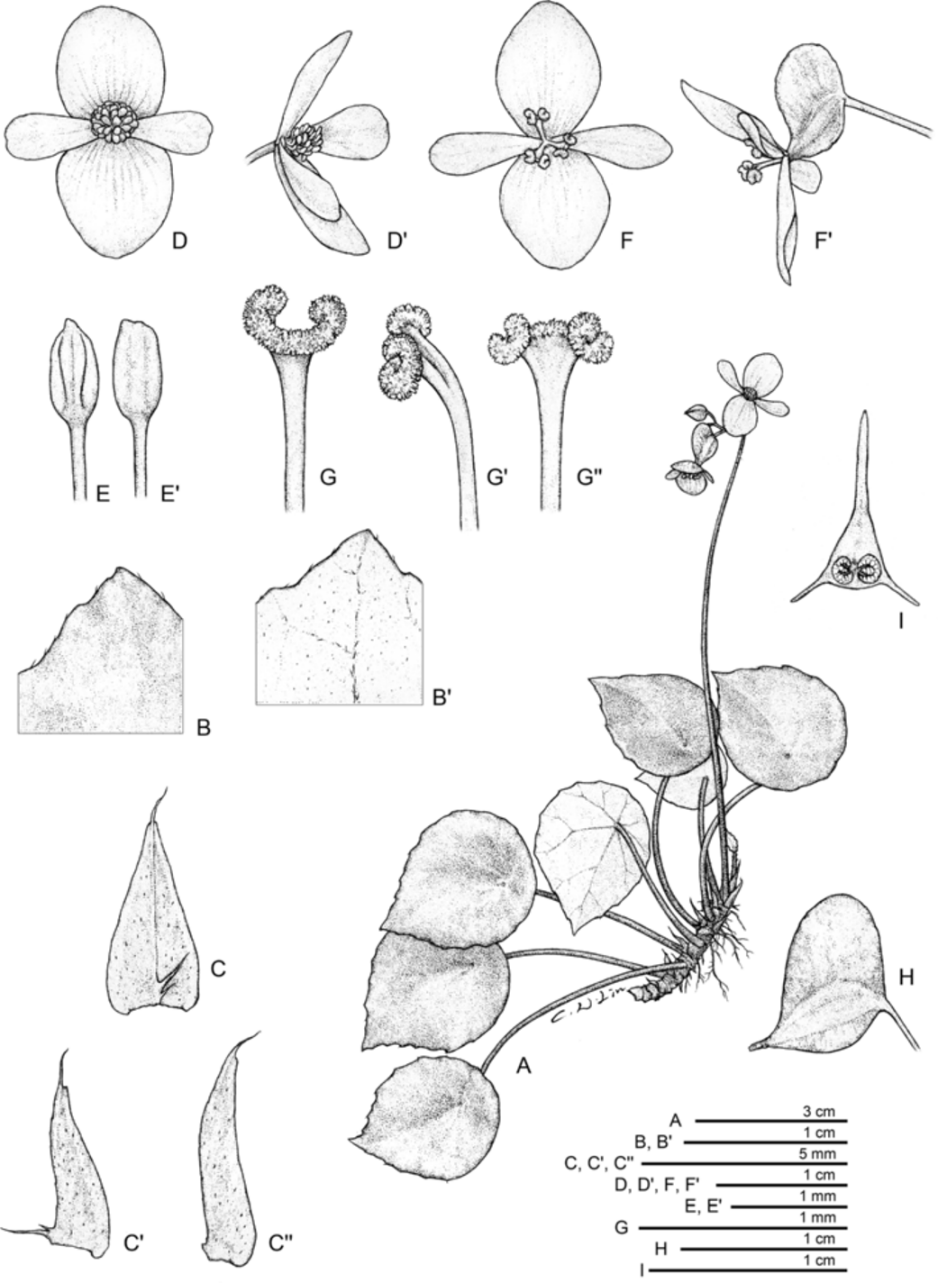
Begonia moneta C.-I Peng, Rimi & C. W. Lin. A. Habit; B. Portion of leaf adaxial surface; **B**’. Portion of leaf abaxial surface; **C**. Stipule, abaxial view, **C**’, **C**”. Stipules, side views; **D**, **D**’. Staminate flower; **E**, **E**’. Stamens, adaxial and abaxial views; **F**, **F**’. Pistillate flower; **G**, **G**’, **G**”. Style and stigmatic band, dorsal, side, and ventral views; **H**. Fruit; **I**. Cross sections of immature fruit. All from *Peng 22344* (HAST).

**Figure 2:**
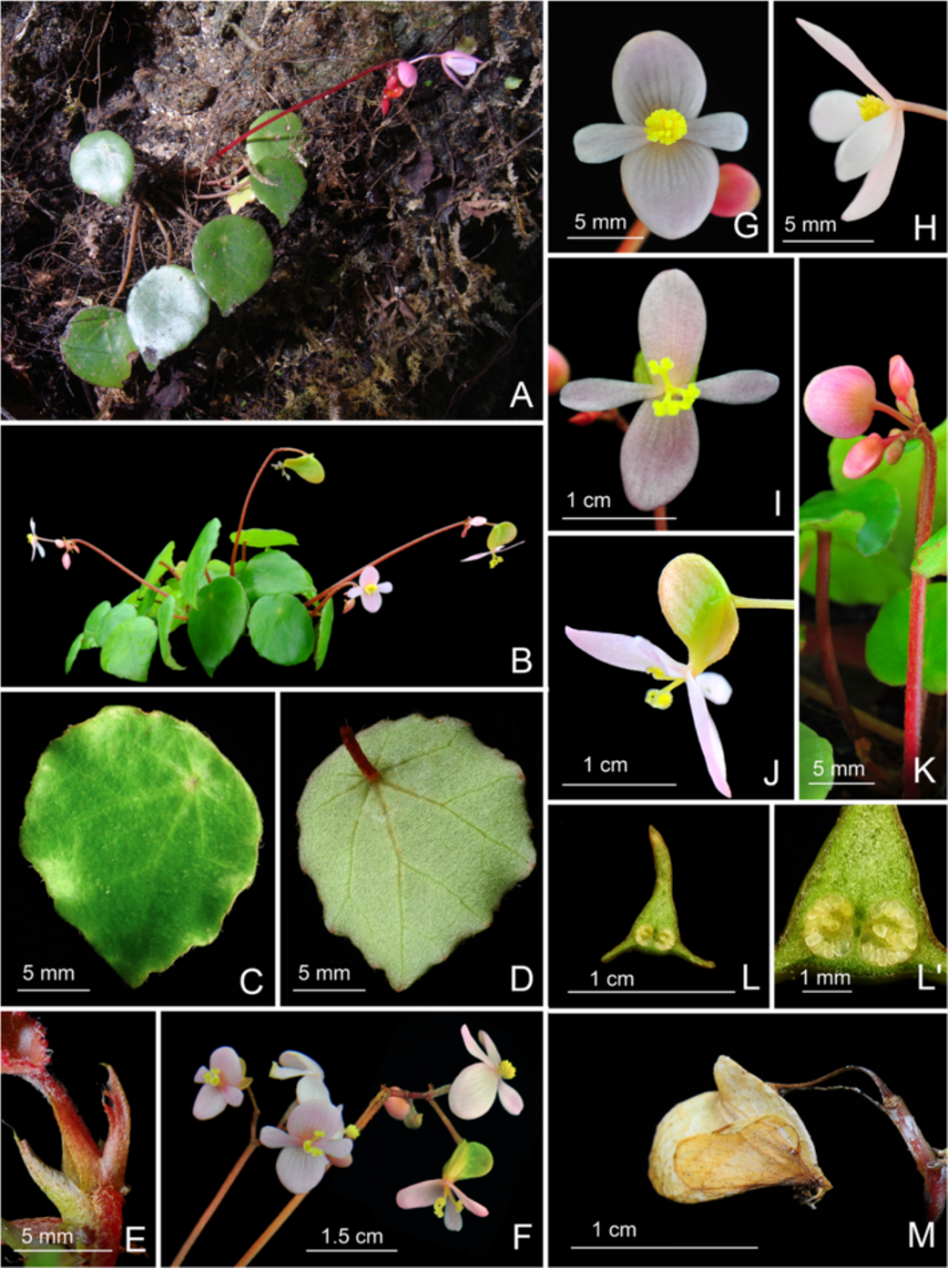
Begonia moneta C.-I Peng, Rimi & C. W. Lin. A. Habit and habitat; **B**. Habit (in cultivation); **C**. Leaf, adaxial surface; **D**. Leaf, abaxial surface; **E**. Stipule on rhizome; **F**. Inflorescence; **G**, **H**. Staminate flower; **I**, **J**. Pistillate flowers; **K**. Young inflorescence with buds; **L**, **L**’. Cross sections of an immature fruit; **M**. Fruit. All from *Peng 22344* (HAST).

Plant monoecious, epipetric, creeping, perennial. **Rhizome** succulent, 3.5–9 cm long, 0.3–0.4 cm across, internodes 0.2–0.6 cm, greenish to reddish brown, subglabrous. **Stipules** pale greenish, triangular-ovate, up to *ca.* 0.7 cm long, 0.3 cm wide, herbaceous, abaxially minutely appressed brown-hairy, entire, strongly keeled on abaxial midrib, apex aristate, arista *ca.* 0.15 cm long. **Leaves** 3–8, simple, peltate, petiole attachment displaced to one side, broadly ovate to orbicular, occasionally angular, base oblique, margin slightly undulate to sparsely obscurely denticulate, apex acuminate to shortly acute, 2.5–4.1 cm long, 1.9–3.8 cm wide, broad side to 2.1 cm wide, adaxially lime green, distinctly fleshy, glossy, glabrous (minutely puberulous on young leaves, hairs caducous), margin sparsely pilosulous, abaxially pale green, sparsely pilosulous on veins; venation basally 7–8 palmate, veins pinnate along primary veins, with 1–3 secondary veins on each side, these branching dichotomously or nearly so, tertiary veins weakly percurrent or reticulate. **Petioles** greenish to reddish, terete, 1.5–2.5 mm thick, 2.2–4 cm long in upper leaves, to 6.5 cm in lower leaves, pilose. **Inflorescence** reddish to crimson, compound cymose, bisexual, axillary, 1–2 arising directly from rhizome, sparsely pilose, erect, with up to 4 orders of branching, exceeding leaves; peduncles 6.5–10 cm long, 0.1–0.2 cm thick; flowers 3–6 per cyme. **Bracts** reddish, herbaceous, ovate to broadly ovate, apex obtuse to acute, at first node of inflorescence ca. 2.5 mm long, 2 mm wide, upper bracts 1.5–2 mm long, 1.2–1.8 mm wide, caducous. **Staminate flower:** tepals 4, margin entire, glabrous, outer 2 oblong to broadly obovate, base rounded, apex obtuse or rounded, 7–10 mm long, 6.5–8 mm wide, abaxially pink, adaxially pinkish to white; inner 2 narrowly ovate to narrowly obovate, base cuneate, apex obtuse to retuse, 6–8.5 mm long, 3–4.5 mm wide, pinkish or whitish; androecium actinomorphic, stamens 13–15, golf club-shaped, slightly compressed, anthers 2-locular, 0.7–1 mm long, yellow, filaments *ca.* 1 mm long, shortly fused at base. **Pistillate flower:** tepals 4, margin entire, glabrous, outer 2 adaxially white to pink, abaxially magenta to white, oblong to widely ovate, apex obtuse or rounded, 7–11 mm long, 6–8 mm wide, inner 2 pinkish or whitish, obovate, base cuneate, apex obtuse to retuse, 6.5–8.5 mm long, 2.5–3.5 mm wide; ovary trigonous-lanceolate, *ca.* 6.5 mm long, 3.5 mm across (wings excluded), pale green to pinkish, 3-winged, wings unequal, crescent-shaped, apically obtuse, longer wing 5–6.5 mm wide, shorter wings *ca.* 2.5 mm wide; locules 2, placentae 2 per locule; styles 3, fused at base, yellow, *ca.* 3 mm long, apically split and C-shaped; stigmas in a spiral band and papillose all around. **Fruit** pendent on a fine stalk 7–10 mm long, *ca.* 7 mm long, 2 mm across (wings excluded), wings crescent-shaped, unequal, 3–6 mm tall.

#### Leaf anatomy

**Figure 3:**
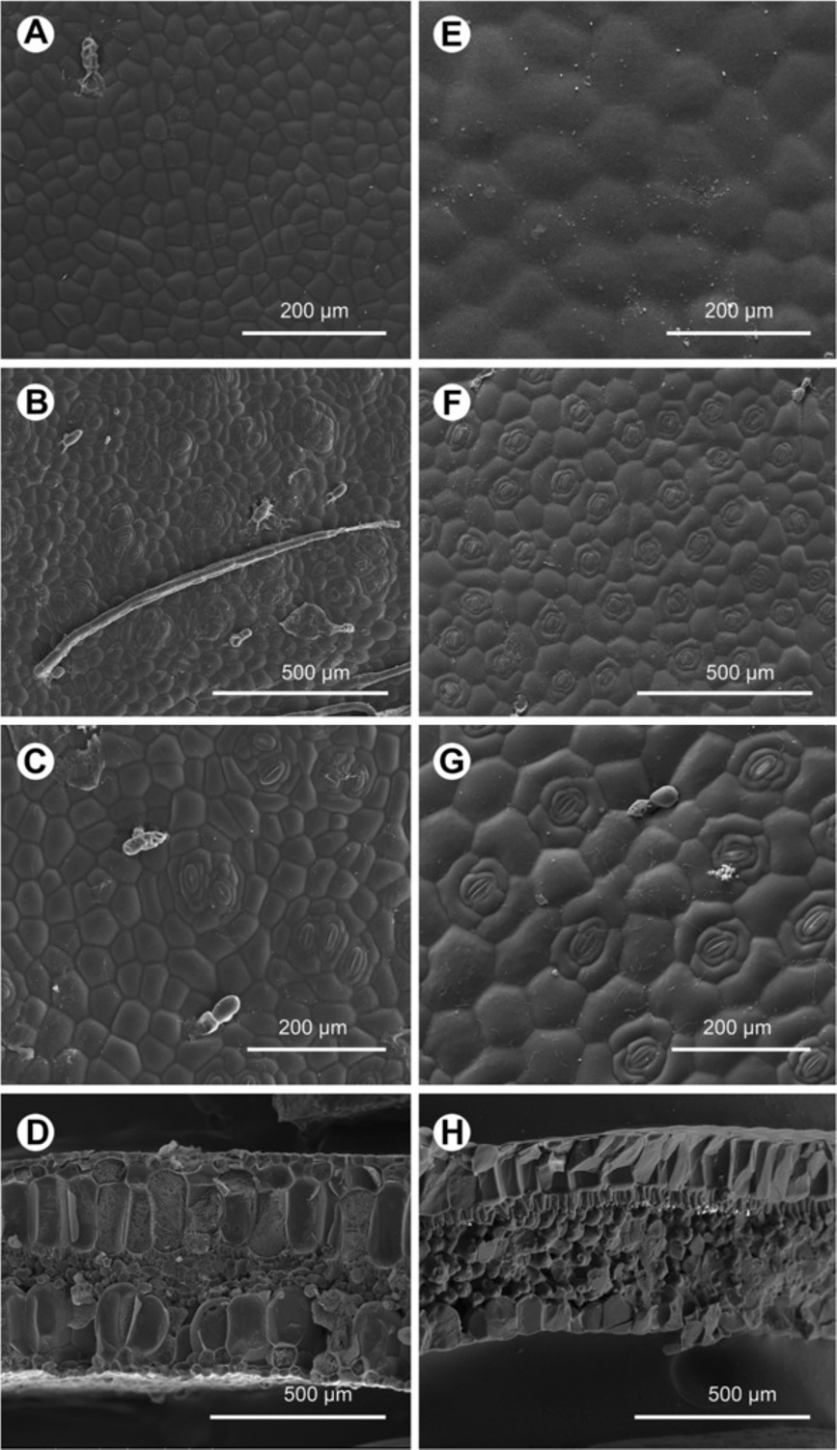
Cryo SEM microphotographs of Begonia leaves. **A**, **E**: Adaxial surface; **B**, **F**: Abaxaial surface; **C**, **G**: stomata complex; **D**, **H**: Cross section. **A**-**D**: *B. moneta* (*Peng et al. 22344*, HAST); **E**-**H**: *B. peridoticola* (*Peng 22343*, HAST)*.*

Adaxial surface glabrous or with sparse, minute glandular hairs (Figure 3A). Cross section ca. 610 μm thick; epidermis single-layered on both surfaces, ca. 23–35 μm thick; hypodermis 2-layered on both surfaces, first-layer 70–85 μm thick, second-layer 180–240 μm thick; palisade tissue 1-cell layered, cells funnel-shaped, ca. 35 μm long; spongy tissue ca. 100 μm long, 4–6 cell-layered (Figure 3D). Abaxial surface with sparse, minute glandular hairs and multiseriate trichomes ca. 1–1.2 mm long (Figure 3B). Stomata complexes clustered (Figure 3B, C).

#### Chromosome cytology

**Figure 4:**
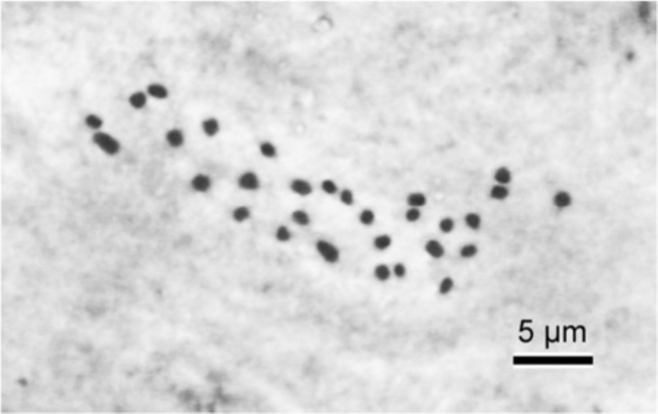
Somatic chromosomes at metaphase of Begonia moneta (2*n* = 30; *Peng 22344*, HAST).

Somatic chromosomes at mitotic metaphase of *Begonia moneta* were determined to be 2*n* = 30 (Figure 4). Among the 30 chromosomes, two are comparatively longer (1.8–2.0 μm long) than the rest; the remaining 28 chromosomes gradually varied from 0.9 to 1.5 μm long. Several longer chromosomes were metacentric or submetacentric, however, centromere positions of shorter chromosomes could not be determined. Satellites were not observed.

In *Begonia* sect. *Baryandra*, chromosome numbers were documented for *B. gueritziana* (2*n* = 28), *B. blancii* (2*n* = 30) and *B. suborbiculata* (2*n* = 30) (Hughes et al. [2011]). Our unpublished data also showed that 2*n* = 28 and 30 are major chromosome numbers in this section.

#### Distribution and ecology

**Figure 5:**
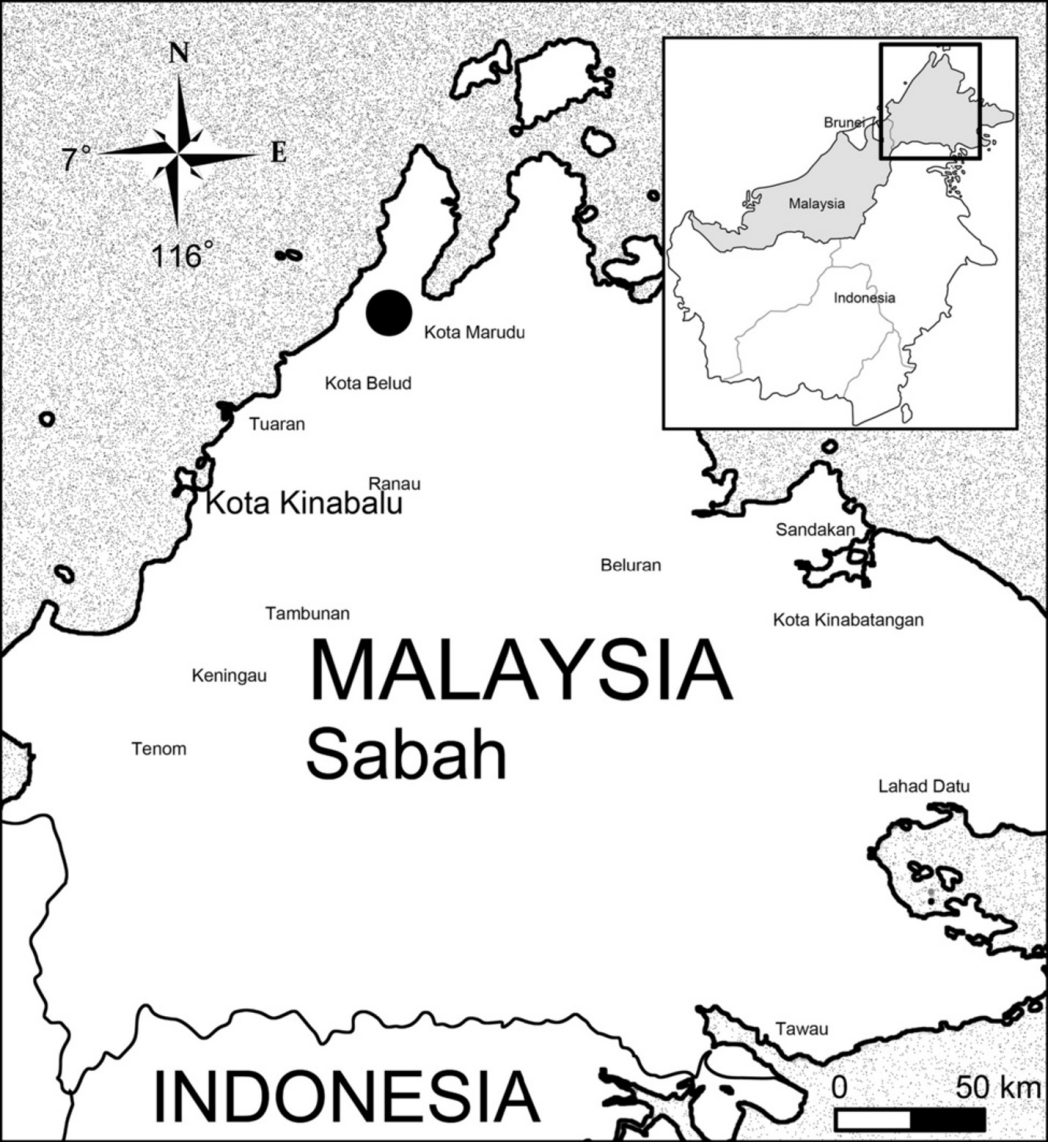
Distribution map of Begonia moneta and B. peridoticola (Solid circle) in Sabah.

MALAYSIA. Borneo. Sabah. Kota Marudu District (Figure 5). Endemic in Kinabalu, growing in an ultramafic area at Serinsim substation, elev. ca. 300–350 m.

#### Etymology

The specific epithet refers to the thick, rounded leaves that are reminiscent of coins.

#### Additional specimen examined

MALAYSIA. Borneo, Sabah. Kota Marudu District: Kinabalu Park–Serinsim substation, Bat Cave Trail, elev. ca.350 m. 13 November 2009, *C.-I Peng 22344,* with *K. F. Chung, W. C. Leong & Rimi Repin* (HAST), same loc., *Yabainus et al. SP 10520* (SNP), *Masrina et al. SP 10538* (SNP), *Masrina et.al SP 10535* (SNP), *Antony van der Ent et. al., SNP 34512* (SNP); Serinsim substation, Mt. Nombuyukong Trail, Plot 1, *Antony van der Ent et .al., SNP 34591* (SNP).

#### Notes

*Begonia moneta* (sect. *Baryandra*) is similar to *B. gueritziana* Gibbs, a widespread species of the same section in Borneo, differing in the peltate (vs. basifixed) leaves and the smaller flower parts. Also, their chromosome numbers are different (*B. moneta*, 2*n* = 30; *B. gueritziana*, 2*n* = 28). The peltate and succulent foliage of *B. moneta* is also reminiscent of *B. burttii* Kiew & S. Julia and *B. payung* S. Julia & Kiew, both of sect. *Reichenheimia*, from Sarawak. *Begonia moneta* is distinct from the two species in having branched (vs. entire) placental lamellae. Additionally, *B. moneta* differs from *B. burttii* in having 4 (vs. 5) tepals in pistillate flowers and markedly unequal (vs. equal) fruit wings. *Begonia moneta* differs from *B. payung* in the smaller leaves and conspicuously winged (vs. wingless) capsules. Clustered stomata complexes were observed in *B. moneta* (Figure 3C), which are likely the way in which the species copes with periodical drought or fluctuating environment (Hughes et al. [2011]; Rubite et al. [2014]).

**2.** *Begonia peridoticola* Rimi, C.-I Peng & C. W. Lin, sp. nov. **-TYPE:** MALAYSIA. Borneo. Sabah. Kota Marudu District, Kinabalu Park – Serinsim substation, Bat Cave, elev. ca. 370 m. Erect herb to 60 cm or more; leaves green above, red beneath; rhizomes lacking. Plants sterile when collected on 13 November 2009. Type specimens with flowers/fruits pressed from plants brought back from the field and cultivated in the experimental greenhouse. *Ching-I Peng 22343-A,* with *Kuo-Fang Chung, Wai-Chao Leong & Rimi Repin* (holotype, SNP; isotypes, A, E, HAST, KEP, SAN).

**Figure 6:**
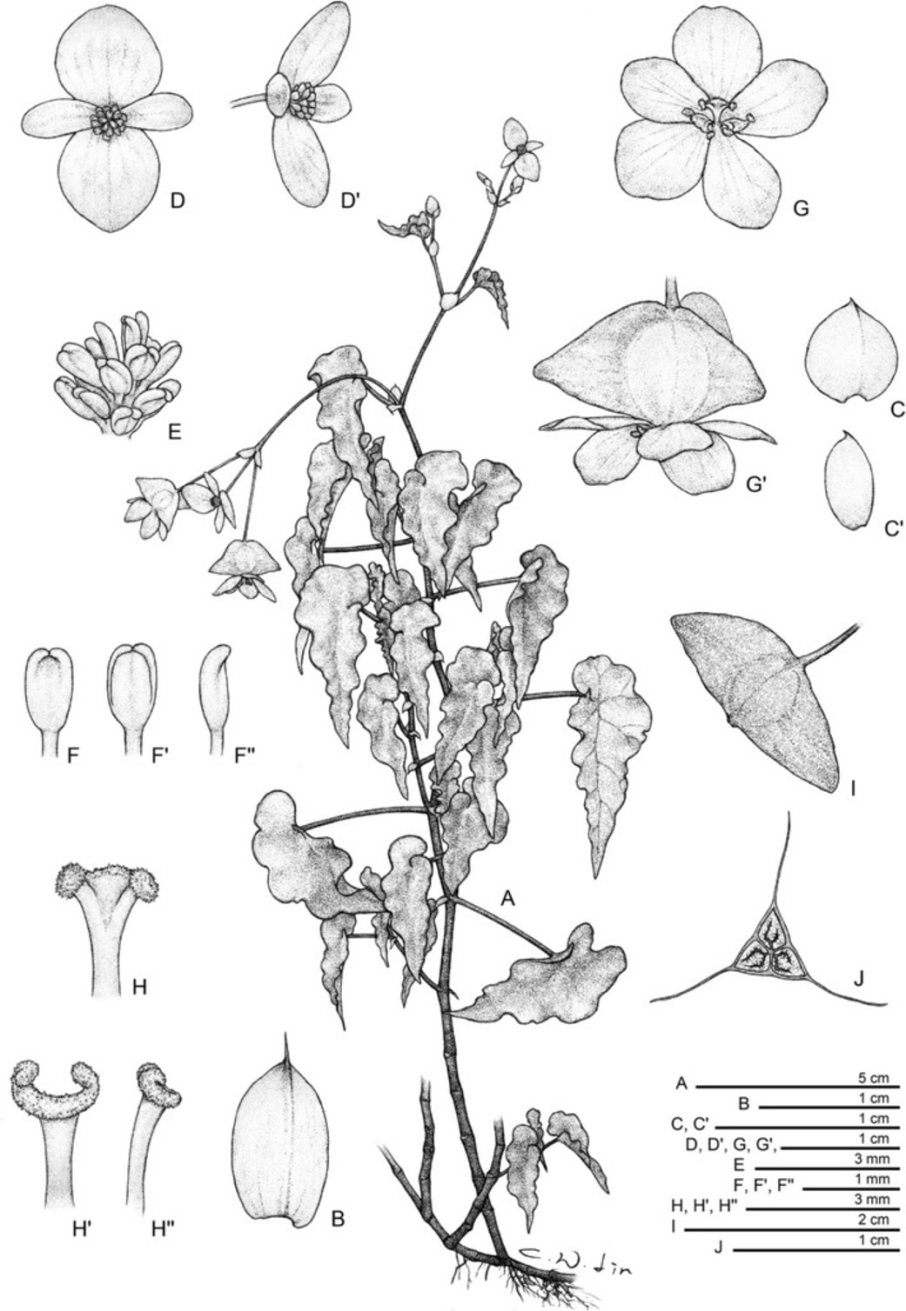
Begonia peridoticola Rimi, C.-I Peng & C. W. Lin. **A**. Habit; **B**. Stipule; **C**, **C**’. Lowermost bract in the inflorescence, face and side views; **D**’, **D**”. Staminate flower; **E**, Androecium; **F**, **F**’, **F**”. Anther, dorsal, ventral and side views; **G**, **G**’. Pistillate flower; **H**, **H**’, **H**”. Style and stigmatic band, dorsal, ventral and side views; **I**. Fruit; **J**. Cross sections of immature fruit. All from *Peng 22343* (HAST).

**Figure 7:**
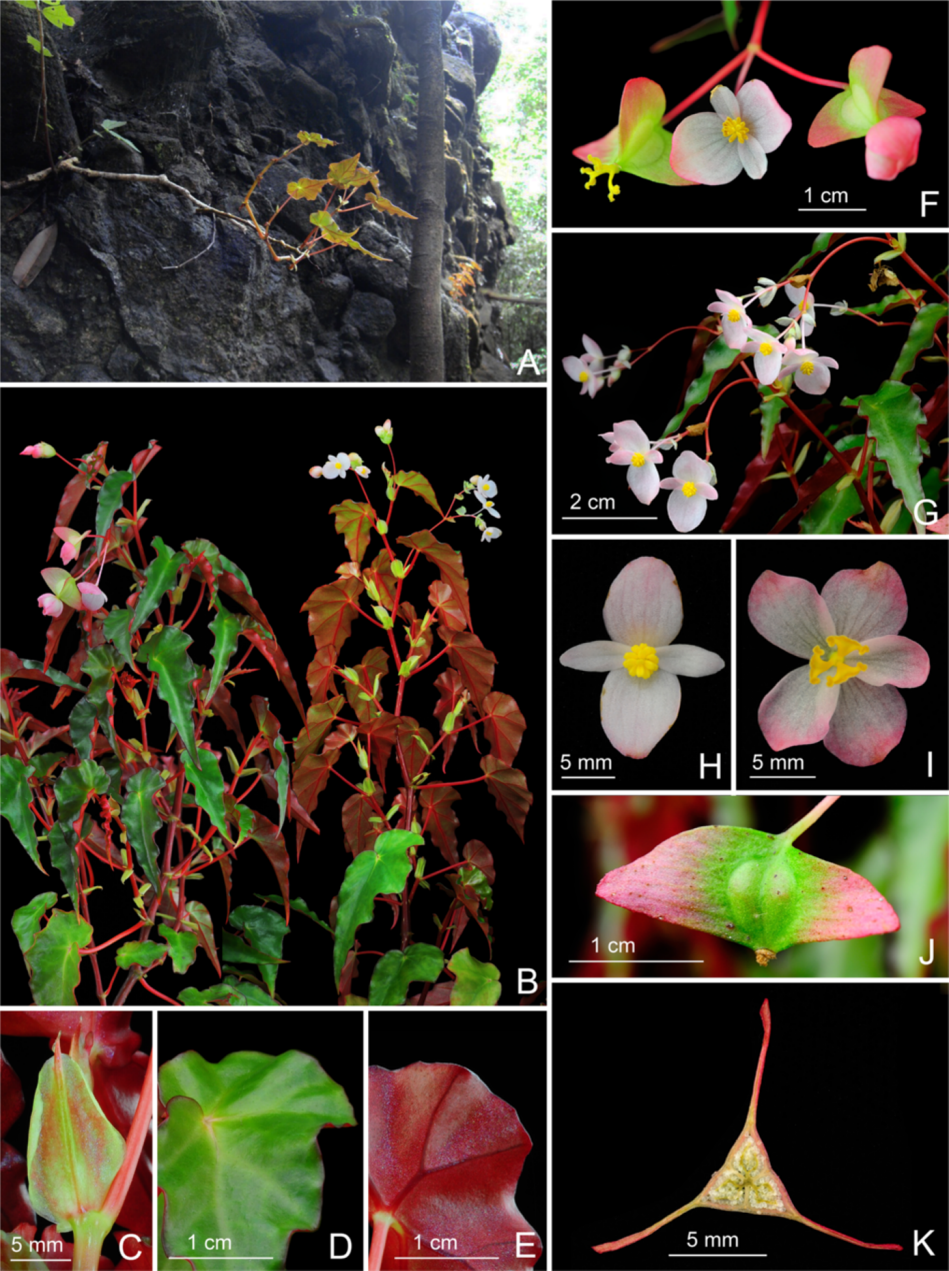
Begonia peridoticola Rimi, C.-I Peng & C. W. Lin. **A**. Habit and habitat; **B**. Habit, dorsal and ventral views; **C**. Stipule; **D**. Portion of leaf, adaxial surface; **E**. Portion leaf, abaxial surface; **F**. Lowermost cyme; **G**. Upper cymes with staminate flowers; **H**. Staminate flower; **I**. Pistillate flower; **J**. Fruit, side view; **K**. Cross section of an immature fruit. All from *Peng 22343* (HAST).

橄欖岩秋海棠 Figures 6 and 7.

Plant monoecious, epipetric, perennial, cane-like. **Stem** crimson to olive green, 30–50 (−70) cm tall, 3–5 mm thick, glabrous, internodes 1.2–4.5 cm. **Stipules** persistent**,** pale green to reddish, broadly ovate-triangular, *ca.* 1.5 cm long, 1 cm wide, herbaceous, ridge keeled, margin entire, apex aristate, arista *ca.* 0.2 cm. **Leaves** numerous, simple, asymmetric, lanceolate-ovate, basifixed, asymmetric with a well-developed basal lobe on one side giving a cordate appearance, margin subentire, strongly undulate, 3.2–7.5 cm long (basal lobes included), 1.7–3.4 cm wide, broad side to 1.9 cm wide, apex acuminate, base markedly unequal, basal lobes cordate, 0.8–1.7 cm long, adaxially malachite green to olive green, margin reddish, glossy, succulent, veins inconspicuous; abaxially crimson to reddish pale-green, glabrous, venation conspicuous, dark red, slightly prominent, primary veins distinguishable, 2.4–5.8 cm long, veins pinnate along primary veins, with 2–3 secondary veins on each side, branching dichotomously or nearly so, tertiary veins weakly percurrent. **Petiole** crimson to olive green, terete, 0.15–0.25 cm thick,1.8–2.6 cm long in upper leaves, to 7.1 cm in lower leaves, glabrous. **Bracts** deciduous, pale green, at first node of inflorescence broadly ovate to orbicular, *ca.* 6 mm long, 5.5 mm wide, margin entire, apex shortly aristate; at summit of inflorescence 3.8–5 mm long, 3.5–4.8 mm wide. **Inflorescence** bisexual, protogynous, axillary in upper axils, reddish, glabrous; lowermost cyme pendant, to 6 cm long, comprising 2 pistillate flowers with or without a staminate flower in-between; second from the lowermost cyme usually with 1 pistillate and 1 staminate flowers; upper cymes staminate, ascending, 3- or 4-flowered. **Staminate flower:** pedicel to 2.2 cm long, glabrous, tepals 4, white to pink, margin entire, apex rounded, glabrous, outer 2 broadly ovate, 7.5–10 mm long, 6.5–8 mm wide, those at lowermost cyme larger, 8–12 mm long, 7.5–10 mm wide, inner 2 narrowly ovate to narrowly obovate, 6–7.5 mm long, 2.8–3.2 mm wide , those at lowermost cyme 8–12 mm long, 7.5–8.5 mm wide; androecium symmetric, stamens *ca.* 25, anthers yellow, oblong-obovoid, slightly compressed, apex retuse, *ca.* 1 mm long, filaments ca. 0.3 mm long, slightly fused at base. **Pistillate flower:** pedicels 19–23 mm long, glabrous; tepals 5, white to pink, margin entire, glabrous, outer 2 broadly ovate to orbicular, 6.5–8.5 mm long, 5.8–8 mm wide, inner 3 broadly obovate, base cuneate, apex obtuse or rounded, 6–9 mm long, 3.5–6.5 mm wide, ovary trigonous-ellipsoid, 8–9 mm long, 6.5–7 mm across (wings excluded), 3-locular, placentation axile, placentae bifid; 3-winged, wings subequal, subtriangular, 9–11 mm long, 8–9.5 mm wide; styles 3, golden yellow, bifid, *ca.* 2.5 mm long, apically split and C-shaped; stigmas in a spiral band and papillose all around. **Fruit** pendent on stalk 5–10 mm long, capsule body 7–9 mm long, 5 mm across, glabrous, wings 3, subequal, subtriangular, margin subentire, 8–11 mm wide.

#### Leaf anatomy

Adaxial surface glabrous (Figure 3E). Cross section ca. 580 μm thick; upper epidermis single-layered, ca. 150 μm thick; lower epeidermis 80–130 μm; palisade tissue 1-cell layered, cells funnel-shaped, ca. 25–55 μm long; spongy tissue ca. 250 μm long, 5–6 cell-layered (Figure 3H). Abaxial surface with sparse, minute glandular hairs. Stomata complexes single, helicocytic (Figure 3F, G).

#### Distribution and ecology

MALAYSIA. Borneo. Sabah. Kota Marudu District. Endemic in Kinabalu Park, elev. ca. 300–400 m, growing on the crevices of peridotite breccia rock face in an ultramafic area.

#### Etymology

The specific epithet is derived from the peridotite breccia rock face at the Bat Cave cliffs near Serinsim sub-station from where it was collected.

#### Additional specimen examined

MALAYSIA. Borneo, Sabah, Kota Marudu District: Kinabalu Park – Serinsim substation, Bat Cave, ca. 370 m elevation. Erect herb, to 60 cm or more; plant sterile when collected, leaves green above, red beneath, rhizomes lacking. 13 Nov 2009, *C.-I Peng 22343* (HAST); same loc.*, Dolois et al. SP 05965* (SNP), *Masrina et al. SP 10536* (SNP), *Antony van der Ent et al. SNP 31263* (SNP), *Antony van der Ent et al. SNP 31260* (SNP).

#### Notes

*Begonia peridoticola* resembles *B. keithii* Kiew (Kiew [1998]) and *B. punchak* Kiew & S. Julia (Kiew & Julia [2007]) in the cane-like stem and succulent leaves. *Begonia keithii* is endemic to limestone in the Semporna Forest Reserve in southeastern Sabah. The new species is readily distinguishable from *B. keithii* in the undulate, lanceolate-ovate (vs. narrowly lanceolate) leaves and cordate (vs. much prolonged) basal lobes. The new species is also similar to *B. punchak* from limestone areas in Kuching, Sarawak, differing in the persistent (vs. deciduous) stipules; yellow, spiral (vs. crimson, widely U-shaped) styles; and much larger capsular wings (8–11 mm vs. 2–3 mm wide).

## Conclusion

A careful study of the literature, herbarium specimens and living plants, both in the wild and in cultivation, supports the recognition of the two new species. Detailed descriptions, line drawings, color plates, chromosome data, foliar SEM observations and comparisons with phenetically similar species are provided to aid in identification.

## Authors’ original submitted files for images

Below are the links to the authors’ original submitted files for images. Authors’ original file for figure 1 Authors’ original file for figure 2 Authors’ original file for figure 3 Authors’ original file for figure 4 Authors’ original file for figure 5 Authors’ original file for figure 6 Authors’ original file for figure 7
