# Supplementary figures and images for: Two new species of Begonia, B. moneta and B. peridoticola (Begoniaceae) from Sabah, Malaysia
Source: Bot Stud. 2015 Apr 10;56:7. doi: 10.1186/s40529-015-0087-5 (PMC5430353; doi:10.1186/s40529-015-0087-5)

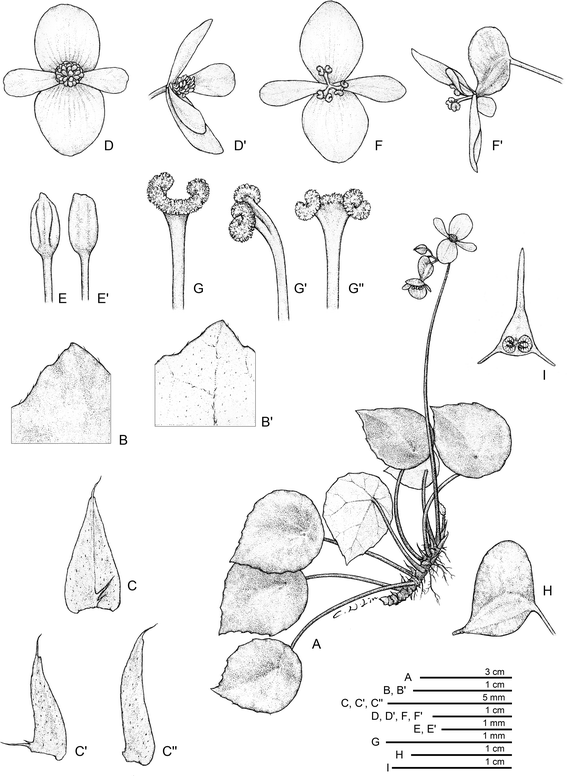

Supplement: Supplementary file 1 — Authors’ original file for figure 1 [file 40529_2015_87_MOESM1_ESM.gif]

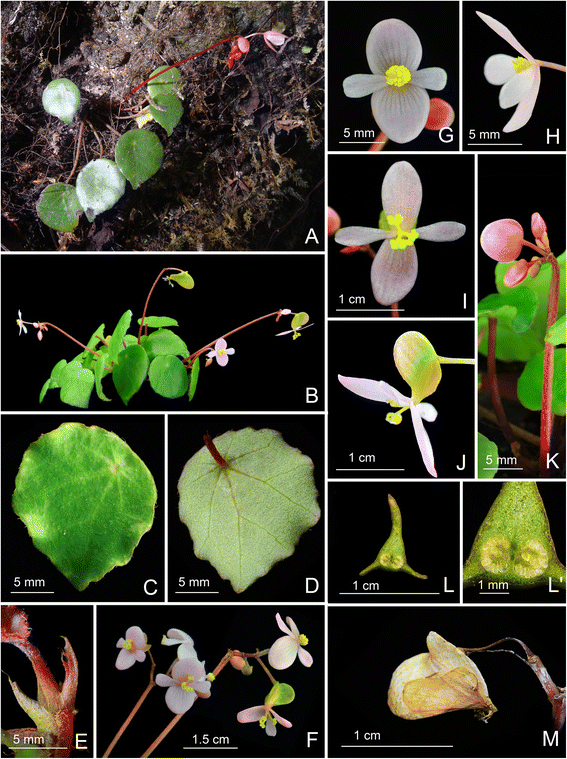

Supplement: Supplementary file 2 — Authors’ original file for figure 2 [file 40529_2015_87_MOESM2_ESM.gif]

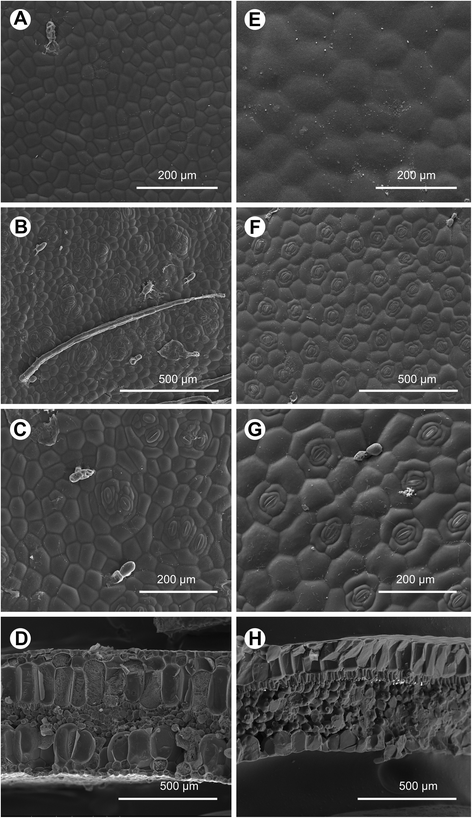

Supplement: Supplementary file 3 — Authors’ original file for figure 3 [file 40529_2015_87_MOESM3_ESM.gif]

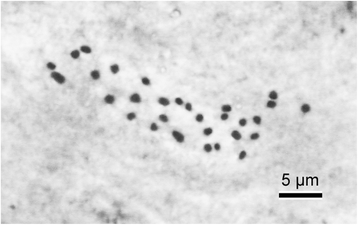

Supplement: Supplementary file 4 — Authors’ original file for figure 4 [file 40529_2015_87_MOESM4_ESM.gif]

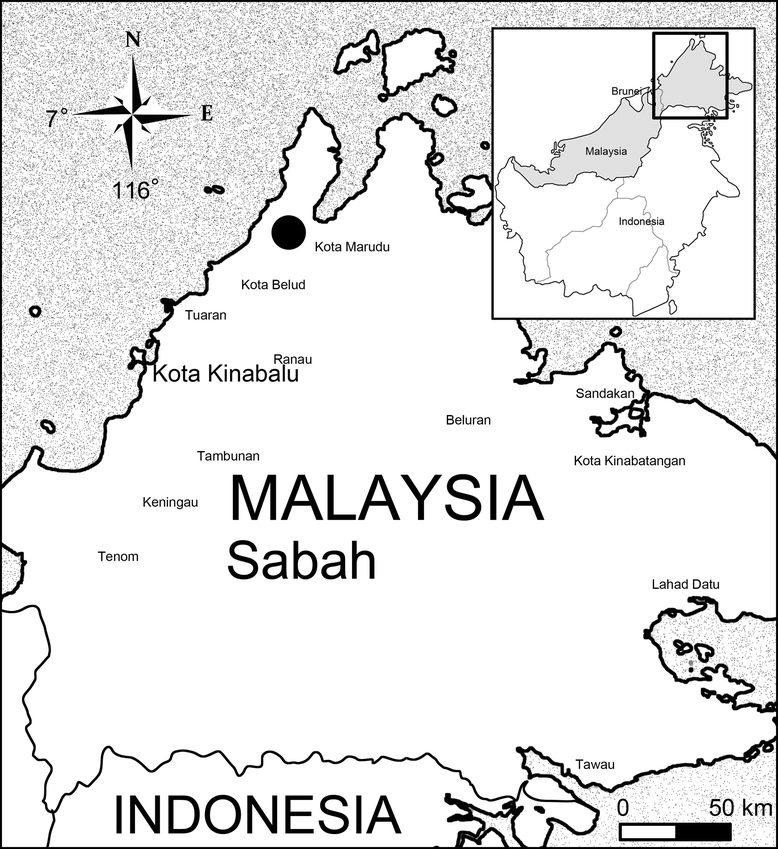

Supplement: Supplementary file 5 — Authors’ original file for figure 5 [file 40529_2015_87_MOESM5_ESM.gif]

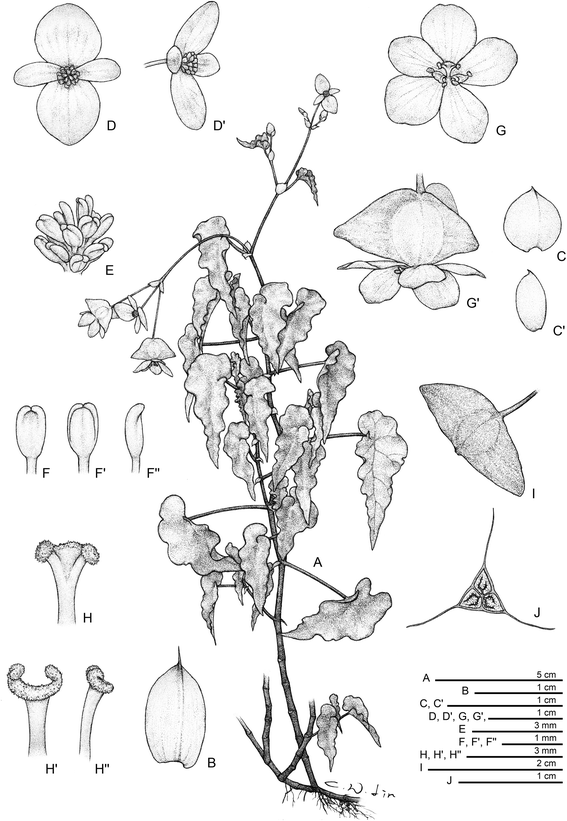

Supplement: Supplementary file 6 — Authors’ original file for figure 6 [file 40529_2015_87_MOESM6_ESM.gif]

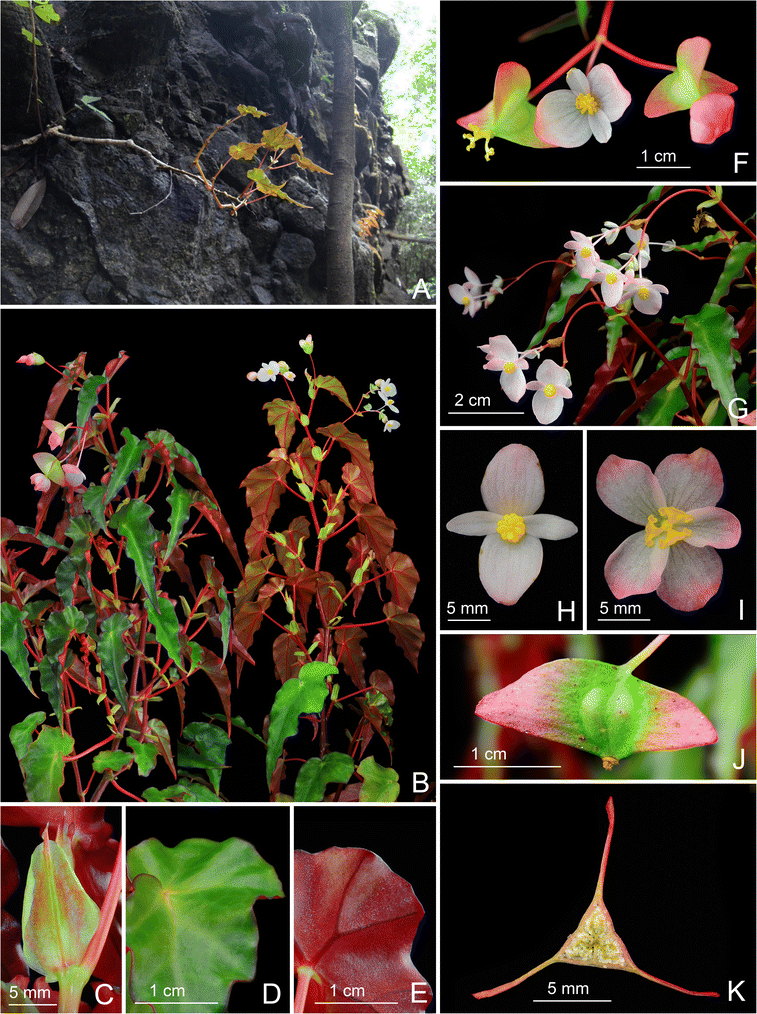

Supplement: Supplementary file 7 — Authors’ original file for figure 7 [file 40529_2015_87_MOESM7_ESM.gif]
